# Tilt engineering of exchange coupling at *G*-type SrMnO_3_/(La,Sr)MnO_3_ interfaces

**DOI:** 10.1038/srep16187

**Published:** 2015-11-04

**Authors:** F. Li, C. Song, Y. Y. Wang, B. Cui, H. J. Mao, J. J. Peng, S. N. Li, G. Y. Wang, F. Pan

**Affiliations:** 1 Key Laboratory of Advanced Materials (MOE), School of Materials Science and Engineering, Tsinghua University, Beijing 100084, China

## Abstract

With the recent realization of hybrid improper ferroelectricity and room-temperature multiferroic by tilt engineering, “functional” octahedral tilting has become a novel concept in multifunctional perovskite oxides, showing great potential for property manipulation and device design. However, the control of magnetism by octahedral tilting has remained a challenging issue. Here a qualitative and quantitative tilt engineering of exchange coupling, one of the magnetic properties, is demonstrated at compensated *G*-type antiferromagnetic/ferromagnetic (SrMnO_3_/La_2/3_Sr_1/3_MnO_3_) interfaces. According to interfacial Hamiltonian, exchange bias (EB) in this system originates from an in-plane antiphase rotation (*a*^−^) in *G*-type antiferromagnetic layer. Based on first-principles calculation, tilt patterns in SrMnO_3_ are artificially designed in experiment with different epitaxial strain and a much stronger EB is attained in the tensile heterostructure than the compressive counterpart. By controlling the magnitude of octahedral tilting, the manipulation of exchange coupling is even performed in a quantitative manner, as expected in the theoretical estimation. This work realized the combination of tilt engineering and exchange coupling, which might be significant for the development of multifunctional materials and antiferromagnetic spintronics.

Perovskite oxides, a class of typical multifunctional materials, have continuously drawn considerable interest of scientific research, due to great variability and various combination of properties, such as giant magnetoresistance^1^,^2^, multiferroics^3^,^4^, and photovoltaic effect^5^,^6^, showing tremendous potential for application in fields of information and energy. By tracing the origin of these abundant phenomena, it is the interactions between orbital, charge, spin and lattice degrees of freedom that lie at the core of these correlated oxides. As almost a ubiquitous structural factor in perovskite oxides, oxygen octahedral tilting has received much attention when combined with other degrees of freedom for property manipulation, e.g. band gap^7^, interfacial conductivity^8^, ferroelectricity^9^ and spin-state transition^10^. Recently, with the experimental realization of hybrid improper ferroelectricity and room-temperature multiferroic materials by tilt engineering, the novel concept of “functional” octahedral rotation shows its significance for the design of multifunctional materials^11^,^12^,^13^. Compared to the relatively mature manipulation of ferroelectricity, tilt engineering of magnetism, especially the exchange coupling, has been little demonstrated, although the successful octahedral control of magnetic transition has been realized^14^.

In this work, we will demonstrate tilt-controllable magnetic properties in a qualitative and quantitative way at the *G*-type antiferromagnetic/ferromagnetic (AFM/FM) interface, in view of the exchange coupling correlated closely to octahedral tilting via Dzyaloshinskii-Moriya (DM) interaction^15^,^16^, which would achieve the combination of tilt engineering with exchange coupling and help the development of multifunctional materials and AFM spintronics.

## Results

### DM interaction as a bridge between octahedral tilting and exchange bias

**Figure 1:**
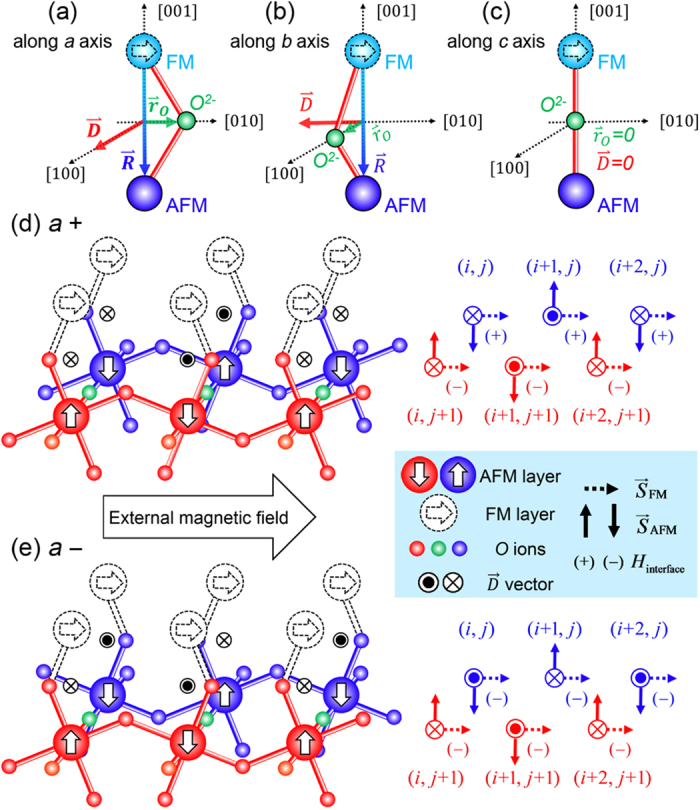
Illustration of DM vector for different tilt patterns. Schematics of the DM vector correspond to the case of tilting axis along (**a**) [100], (**b**) [010] and (**c**) [001]. 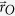 denotes the displacement of the oxygen ion and 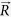 is a vector pointing from FM atom to AFM atom. Sketches of the *G*-AFM/FM interface are given for (**d**) inphase and (**e**) antiphase tilting about [100] (*a* axis), i.e., *a*^+^ and *a*^−^ respectively, with the sign of 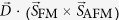 shown in the right simplified diagram for the whole interface. The direction of external magnetic field along [010] is marked with an arrow in the figure.

In order to realize the manipulation of exchange coupling via octahedral tilting, first of all, it is necessary to figure out how octahedral tilting acts on the behavior of exchange bias (EB). As shown in Dong’s work^15^, interfacial Hamiltonian of spin-spin interaction for AFM/FM bilayers can be written as:


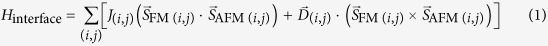


where 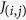 and 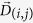 denote the coefficient of superexchange coupling and the DM vector respectively^17^, while 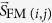 and 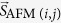 parameter spin vectors in FM and AFM layers, with the site index of (*i, j*). A negative 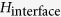 implies a favorable state in energy with effective AFM/FM coupling, leading to the emergence of EB. Here we only consider the contribution of the Mn-O-Mn bonds across the interface to 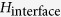, because the DM interaction term equals to zero for in-plane bonds with all the magnetic moments collinear, i.e. the adjacent magnetic moments are parallel to each other in the same FM plane while they are antiparallel in the same AFM plane. For the perovskite AMO_3_, 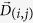 is parallel to 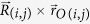 (Fig. 1), with the magnitude estimated by the linear form^18^:


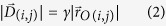


where 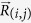 is the direction of M-O-M bond and 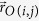 is the off-bond displacement of oxygen ions, while *γ* is a constant varying for different materials.

Considering the staggered configuration of 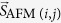 for *G*-AFM, superexchange coupling for adjacent sites counteracts with each other and causes no EB. However, the situation changes dramatically for DM interaction due to the possibly simultaneous alternation of 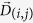 with 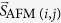 relevant to different atomic arrangement, i.e. different tilt pattern, which has received little attention in previous work^15^. This provides the origin of our idea for the manipulation of exchange coupling by octahedral tilting.

As displayed in Fig. 1a, a tilting about [100] (*a* axis) causes a displacement 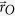 along [010] (*b* axis) and thus 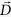 points to [100]. The situation is analogous for *b*-axis tilting (Fig. 1b). Nevertheless, for the case of *b*-axis tilting, with the magnetic field fixed along [010] in our work, 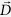 is collinear with 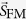 and would trigger no EB for a zero DM interaction term according to Equation (1). We also note that if the tilting axis is along [001] (*c* axis), nothing would be contributed to interfacial DM interaction in that no off-bond displacement 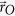 exists as shown in Fig. 1c. It is then concluded that only in the case of *a*-axis tilting, DM interaction can be effective for inducing an EB. In this situation, 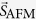 along *c* axis is greatly favored for the largest negative DM interaction term of 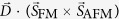.

In general, octahedrons in adjacent planes can rotate in a same or opposite direction, as marked by the superscript of the Glazer notation, like ‘*a*^+^’ or ‘*a*^−^’, which is defined as an inphase or antiphase tilting respectively^19^. For a clear illustration, the tilting axis is donated by the base letter (*a, b* and *c*) in this paper. As can be seen in Fig. 1d,e, although the 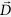 vector seems to counteract with each other for adjacent bonds in the same *b*-*c* plane, the synchronous alternation of 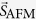 leads to the accumulation of 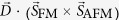. Then we note that in Fig. 1d, the inphase rotation (*a*^+^) causes a consistent DM vector in adjacent planes, i.e. the front plane (red) and the back plane (blue). On contrary, an alternative DM vector is induced by the antiphase rotation (*a*^−^) for the adjacent plane in Fig. 1e. For the case of *a*^−^ tilting, a combination of the alternative 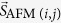 and the simultaneous variation of 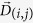 over the whole interface produces a significant negative 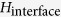 for the accumulation of each negative DM interaction term, while *a*^+^ tilting causes no EB due to the counteraction of DM interaction in two adjacent planes, as can be seen from the corresponding sign of 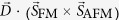 illustrated in the right sketches. Thus, theoretically, only the *a*^−^ tilting contributes to the EB effect and if *a*^−^ is the major tilt pattern in AFM layer, a remarkable EB would appear at the FM-AFM interface predictably.

### Strain manipulation of tilt patterns according to first-principles calculation

Now the question comes to the acquirement and manipulation of a certain tilt pattern. Given that octahedral tilting originates from the tendency of space relaxation^20^, epitaxial strain could be an effective manner for the control of tilt patterns. To reveal the strain effect on octahedral tilting, we perform first-principles calculation for different strained SrMnO_3_ (SMO), a typical *G*-AFM perovskite with a Néel temperature of 233^−^260 K^21^,^22^. (Supplementary for more details of calculations) Structure optimization discloses that the bulk SMO has a cubic lattice constant of 3.83 Å with an atomic magnetic moment of 2.53 μ_B_, which is close to the experimental data^22^. To introduce the strain effect, we set the in-plane lattice constant *a* = *b* at a series of values around the bulk lattice and keep the lattice volume constant as the bulk one. To ensure the validity of our model during the manipulation, it is verified that SMO maintains a *G*-AFM ground state as the in-plane lattice constant varies from 3.78 Å to 3.90 Å (Supplementary Fig. S1), corresponding to two common substrates, i.e. LaAlO_3_ (LAO) and SrTiO_3_ (STO) respectively, which are used in our study later.

**Figure 2:**
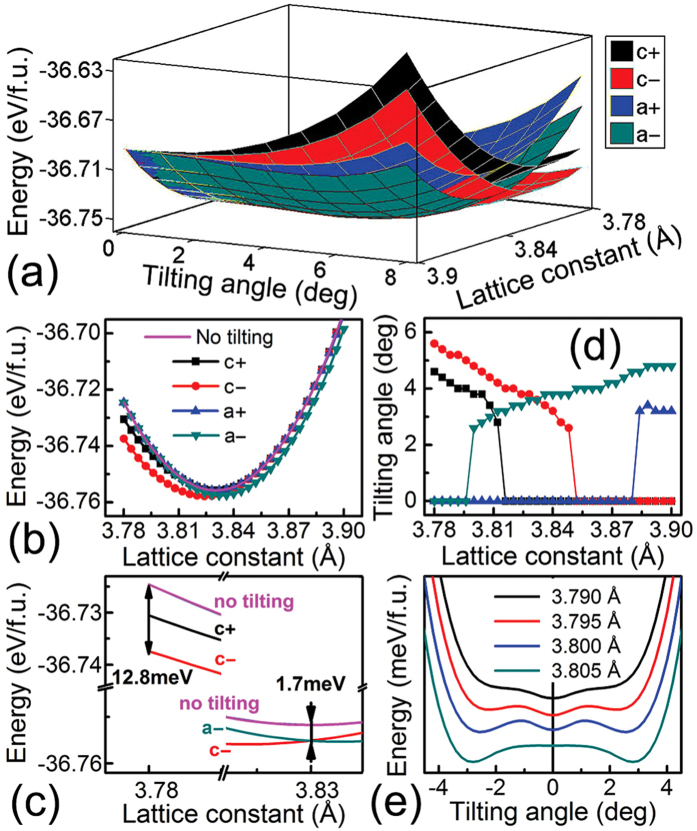
Dependence of SMO energy on tilting angle and in-plane constraint. (**a**) Dependence of total free energy per formula unit on tilting angle and in-plane lattice constant for tilting modes of *c*^+^, *c*^−^, *a*^+^ and *a*^−^. (**b**) Minimum total free energy per formula unit as a function of in-plane lattice constant for different tilting modes with the no-tilting case marked by the solid line. (**c**) Partial enlarged view of the energy curve around the lattice constant of 3.78 Å and 3.83 Å. (**d**) Dependence of the tilting angle of minimum energy on in-plane lattice constant. (**e**) Relative change of the two valleys on the energy curve of *a*^−^ tilting with the change of the in-plane lattice constant.

Then we turn to the strain effect on octahedral tilting. Total free energy per formula unit for uniaxial tilting is presented in Fig. 2a as functions of in-plane lattice constant and tilting angle for different tilting modes, where adjacent oxygen octahedrons along the axes of *a* and *c* rotate in the same (*a*^+^ and *c*^+^) or opposite (*a*^−^ and *c*^−^) direction. The energy surfaces of antiphase tilting, i.e. *a*^−^ and *c*^−^, are lower than their inphase counterparts. To be more specific, Fig. 2b shows the lattice dependence of the lowest energy for these tilting modes. The energy curve of no-tilting system is also plotted as a reference. There appears a crossover in energy as SMO is relaxed to its bulk value of 3.83 Å, accompanied by a preferred rotation of *a*^−^ and *c*^−^ under tensile and compressive strain respectively. This dependence is clearly reflected in Fig. 2c, where details near the bulk parameter are shown for clarity. Although we discuss the strain effect on tilt pattern in uniaxial mode, the results are similar to other perovskite systems taking three-axis rotations into consideration, such as LaAlO_3_^23^. Note that, for the bulk lattice, the energy of no tilting is higher than other tilt patterns, which seems inconsistent with no tilting (*a*^0^*a*^0^*a*^0^) for bulk SMO. This energy deviation could be ascribed to the settings of our model. Nevertheless the energy deviation of ~1.7 meV for the bulk lattice is one order of magnitude smaller than the scenario (~12.8 meV) with large compressive strain (Fig. 2c), where the tilting is clearly preferred when taking the large energy deviation into account. To be noted, tilting angle of each point is the most favorable value in energy. Consequently, curves of other patterns would overlap with the no-tilting one under large strain, because the minimum energy of this tilt pattern is reached at a zero tilting angle for this strain state, meaning that it has the same energy as the no tilting case.

Tilting angle of minimum energy as a function of lattice constant is displayed in Fig. 2d. Within the tensile range, as the in-plane lattice constant shrinks from 3.90 Å to the bulk value of 3.83 Å, the optimal tilting angle of *a*^−^ tilting decreases from 4.8° to 3.8°. A similar decreasing tendency is observed for *c*^−^ tilting with compressive constraint varying from 3.78 Å to the bulk value. Besides, these angles of minimum energy in antiphase cases are greater than their inphase counterparts, supporting the analysis above that antiphase rotation of adjacent octahedrons are much more favorable in energy whatever the tilting axis is. Note that there appears a sudden drop of tilting angle to zero, which can be ascribed to the relative change of two valleys on the energy curve at that in-plane lattice constant. The change of tilting angle associated with the minimum energy is presented in Fig. 2e, where *a*^−^ tilting is taken as an example. As the strain becomes more compressive (from 3.810 Å to 3.795 Å), the energy valley of no tilting becomes lower than the valley of the case with a tilting angle ~3°, resulting in the sudden drop of the tilting angle from ~3° to 0°.

**Figure 3:**
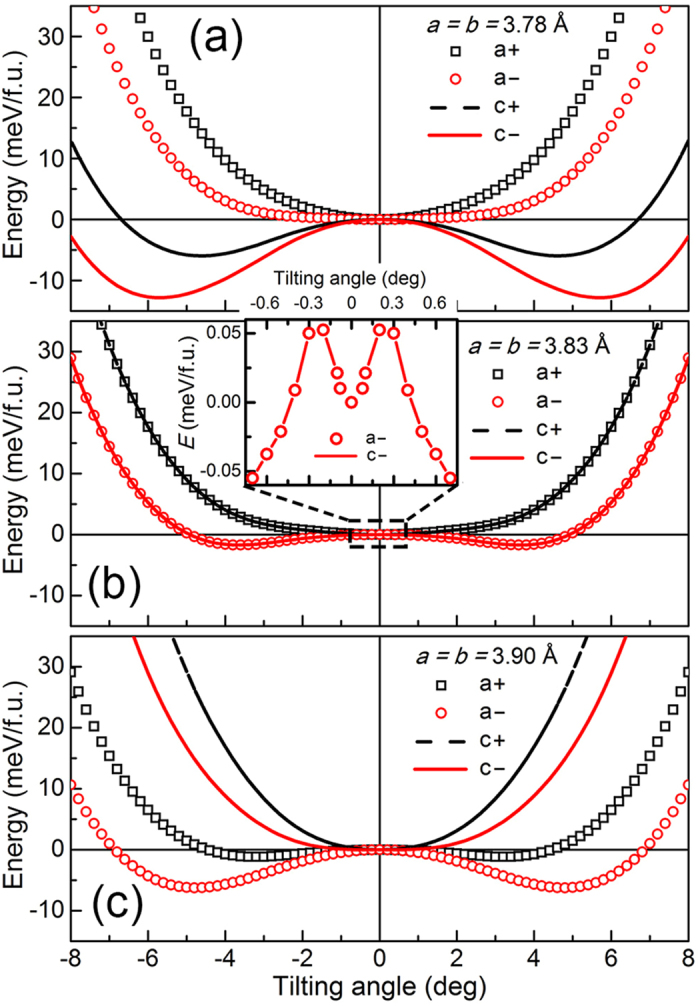
Energy as a function of tilting angle for three selected lattice. The tilting-angle-dependent total free energy (*E*) per formula unit for three specific lattice parameters of 3.78 Å (**a**), 3.83 Å (**b**) and 3.90 Å (**c**), corresponding to the value of LAO, SMO and STO respectively. Energy of no-tilting case is set to zero as a reference. The tilting angle dependent energy for *a*^−^ and *c*^−^ tilting is shown in the expanded scale in the inset of (**b**).

For further understanding, we focus on the tilting-angle dependent energy of these four rotation modes for three specific lattice parameters, i.e., 3.78 Å, 3.83 Å, and 3.90 Å. Relevant data are shown in Fig. 3a–c, respectively, where the energy of the no-tilting case is set to zero as a reference. Due to the octahedral connectivity, a negative tilting angle means a reverse rotation of all the octahedrons, equivalent to a shift of one unit cell along <100> of the whole lattice, which brings no energy change compared to the same positive angle. Thus the curve is symmetric about 0° for the same positive and negative tilting angle. Note that more than one tilting mode exhibits a lower energy than the case without tilting. This implies a coexistence of different rotations, with the relative ratio determined by the energy difference. A closer inspection of Fig. 3a shows that compressive strain in SMO favors the tilting of *c*^−^ and *c*^+^, in stark contrast to almost pure *a*^−^ tilting favored by tensile strain in Fig. 3c. Then we turn to the case with lattice constant of bulk SMO in Fig. 3b. Although there exists a local minimum at 0° as shown in the inset, consistent with the equilibrium structure with no tilting of bulk SMO, the lower and equivalent energy of *a*^−^ and *c*^−^ tilting means that the coexistence of *a*^−^ and *c*^−^ tilting is energetically favorable when the film relaxes to the bulk lattice under the non-equilibrium condition of growth. The overlapping of energy curves of *a*^−^ and *c*^−^ in Fig. 3b reveals that *a*^−^ and *c*^−^ are comparable in energy when the film relaxes to the bulk lattice. Besides, the subtle energy difference between these two modes and the no-tilting case agrees with the fact that no tilting exists for ideal bulk SMO. Interestingly, the energy changes drastically with the variation of tilting angles, especially for *a*^−^ which accounts for the appearance of EB, serving as the pre-requisite for a quantitative modulation of the EB strength via the tilting angle in the vicinity of the *G*-AFM/FM interface, which is discussed in the last section of this paper.

### Design and characterization of octahedral tilt pattern

On the basis of theoretical inferences above, a manipulation of octahedral tilting can be realized by epitaxial strain. A series of SrMnO_3_/La_2/3_Sr_1/3_MnO_3_ (SMO/LSMO) bilayers were designed and prepared on (100)-oriented substrates by pulsed laser deposition (PLD). SMO was deposited on the substrates and then LSMO was deposited on SMO. Thus, the interfacial Mn-O-Mn bonds which would contribute to exchange coupling are determined by the tilt pattern of the bottom SMO due to the imprinting of rotation behavior^24^, despite the bulk *a*^−^*a*^−^*a*^−^ pattern of LSMO^25^. SrTiO_3_ and LaAlO_3_ substrates were selected to offer tensile and compressive strain, respectively. A gradient of SMO thickness was adopted, i.e. 10, 20, 30, and 40 unit cell, to introduce different state of relaxation at the interface, while the thickness of LSMO was kept at 20 unit cell (u.c.). The whole process was monitored by *in situ* RHEED (reflected high-energy electron diffraction). Well-defined oscillations and clear images of spots and stripes (Supplementary Fig. S2) indicate a layer-by-layer growth of the films, offering an ideal interface which meets our model well with the influence of interfacial roughness negligible^26^. It’s verified further by the results of atomic force microscope that the surface roughness of SMO single films is less than 0.3 nm, which is not shown here.

For the first step towards tilt engineering of exchange coupling, characterization of tilt patterns in SMO under different epitaxial strain is given. Since the unit cell would double if octahedral tilting occurs, a Bragg diffraction peak of special half integer index (*H*/2 *K*/2 *L*/2) appears, which is known as the half order Bragg diffraction peak. The index of these peaks shows different characteristics, corresponding to different tilt patterns^27^. For example, an inphase rotation of *a*^+^ causes the doubling of unit cell along the axes of *b* and *c*, leading to the appearance of half order peaks with an even-odd-odd characteristic of (*H K L*). Similarly, *b*^+^ and *c*^+^ can be distinguished by the index feature of odd-even-odd and odd-odd-even respectively. On the other hand, because an antiphase rotation doubles unit cell along all the three axes, the index of (*H K L*) would show a characteristic of odd-odd-odd. Besides, when the rotation occurs about a certain axis, an extra relationship of the index is given by theoretical analysis, e.g. the peak of *a*-axis tilting has an index of *K* ≠ *L* (*H* ≠ *L* for *b*-axis and *H* ≠ *K* for *c*-axis)^27^. Thus, based on the characteristic of the half-order Bragg diffraction, we found different special (*H*/2 *K*/2 *L*/2) diffraction position in reciprocal space of the single-crystal substrates, i.e. STO and LAO, for the detection of different tilt patterns, and then collected the signal of diffraction peak from the upper SMO epitaxial films by varying *L* with *H* and *K* fixed in the *L*-scan mode, to judge the pattern of octahedral tilting in SMO.

**Figure 4:**
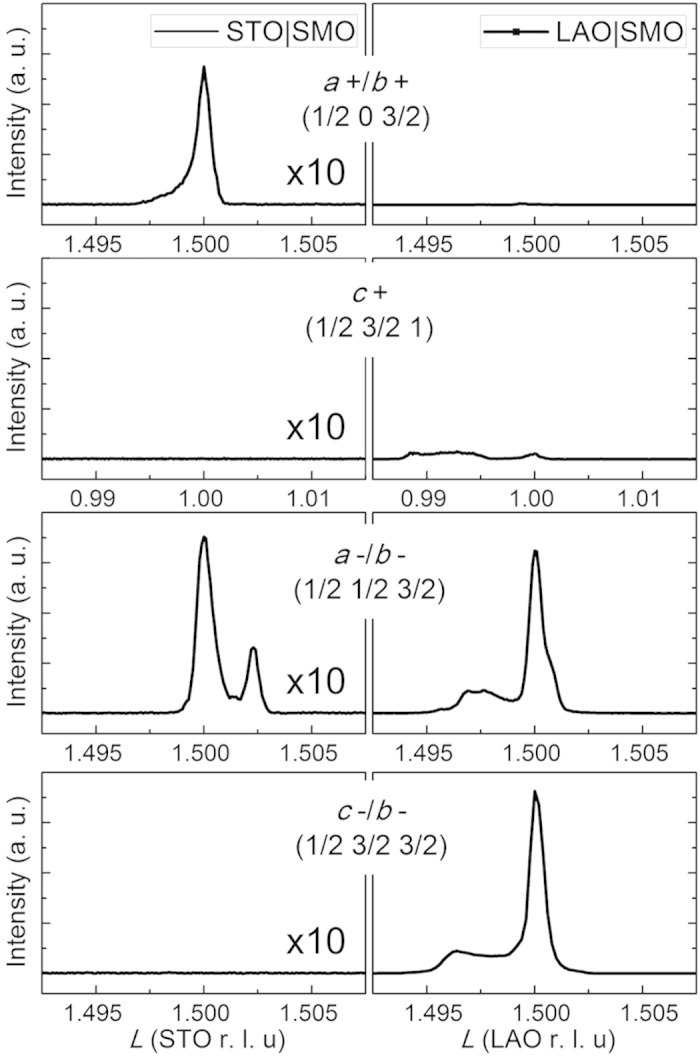
Half order Bragg diffraction of SMO films grown on STO and LAO. Characteristic peaks of half order Bragg diffraction by *L*-scan in reciprocal space for SMO films grown on substrates. Left column shows signals from SMO on STO, while right column shows signals from SMO on LAO. The intensity of the left column is multiplied by ten to be comparable magnitude to that of the right ones.

In order to highlight signals from films, special diffraction peaks of SMO single layers with a thickness of 40 u.c. on STO and LAO substrates were measured, as displayed in Fig. 4. Because the signals of half order Bragg diffraction are gained around the specific (*H*/2 *K*/2 *L*/2) peaks of substrates, peaks of substrates appear at the position of the exact half integer. Note that while SMO on LAO shows strong signals of *a*^−^ and *c*^−^ from substrates (Fig. 4g,h), consistent with the tilt pattern *a*^−^*a*^−^*a*^−^ of LAO, the results of *L*-scan for SMO on STO indicates the existence of in-plane tilting in STO (Fig. 4a,c) despite the pattern *a*^0^*a*^0^*a*^0^ of ideal STO, which is thought to be defect related^28^. This is reasonable taking the low intensity of STO peaks in comparison with their LAO counterparts into account. That is, the peak intensity of STO in the left column becomes comparable to that of LAO in the right column when multiplied by ten, as denoted in this figure.

Then we focus on the signals from SMO. Because SMO has a smaller lattice than STO, peaks of SMO on STO in the left column would appear at a higher *L* value than that of STO, compared to a lower *L* value for SMO on LAO in the right column. Figure 4c presents an extra peak on the right of the peak (1/2 1/2 3/2) from STO, indicating *a*^−^/*b*^−^ rotation in SMO. Differently, concerning signals from *a*^+^/*b*^+^ rotation at (1/2 0 3/2) in Fig. 4a, only the signal of STO is observed, excluding such a rotation in SMO. Meanwhile, *c*^+^ and *c*^−^ rotations are undetectable in SMO and STO with no peaks in Fig. 4b,d. Thus, for SMO on STO, we only observe the signal of *a*^−^/*b*^−^, consistent with that only *a*^−^ is energetically favorable under tensile strain (Fig. 3c). The case differs when using LAO substrate. Besides the peaks of LAO, extra signals from SMO are observed in Fig. 4f–h, for the situation of *c*^+^, *a*^−^/*b*^−^ and *c*^−^ respectively, while *a*^+^/*b*^+^ are still not detected in Fig. 4e. Noteworthily, the peak of *c*^+^ has a much smaller intensity than those of antiphase tilts, indicating a difference in relative proportion. Such a mixture of rotations agrees well with the first-principles calculation for the relaxation of compressive strain (Fig. 3a,b). Thus we verify that different tilt patterns could be attained in experiment via different epitaxial strain, that is, pure *a*^−^ is induced by tensile strain, whereas *a*^−^, *c*^+^ and *c*^−^ coexist in the compressive SMO. Here, the observation of *a*^−^ tilting in compressive SMO confirms that despite the little energy difference between no tilting and *a*^−^ tilting in Fig. 3b, *a*^−^ tilting exists in the real system, which may be attributed to defects introduced during the process of non-equilibrium growth.

### Octahedral tilting dependent exchange bias

**Figure 5:**
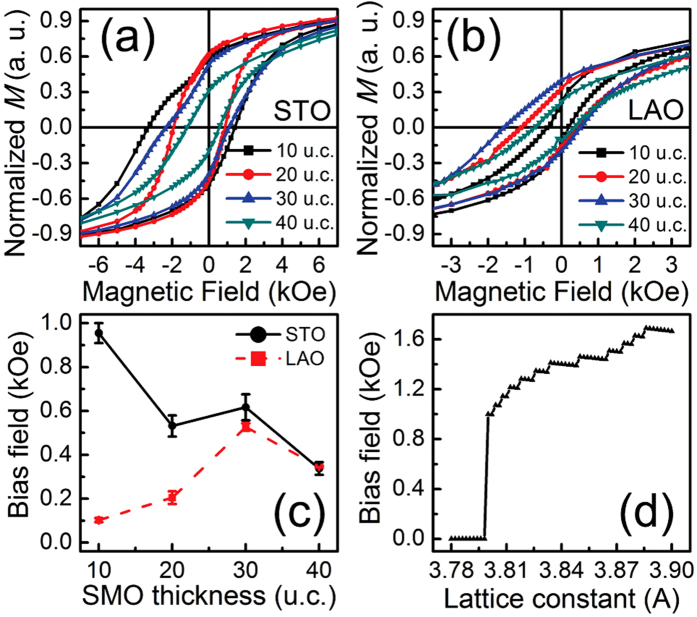
Thickness dependent hysteresis loops of SMO/LSMO bilayers and quantitative estimation. Magnetization loops of SMO (*t*)/LSMO (20 u.c.) on STO (**a**) and LAO (**b**) for a series of SMO thicknesses (*t* = 10, 20, 30, 40 u.c.). (**c**) The bias field as a function of SMO thickness. (**d**) The dependence of calculated bias field on in-plane lattice constant for the uniaxial tilting of *a*^−^.

To see the tilt engineering of magnetic property, Fig. 5a,b show the magnetization loops of different samples recorded by a SQUID (superconducting quantum interference device) magnetometer at 4 K after cooling from 300 K at a field of 3 kOe along [010]. Their saturation fields up to ~15 kOe are not shown to highlight the shift of the loops. With different tilt patterns on different substrates, SMO/LSMO bilayers demonstrate different magnetic properties, just as expected in our model. Remarkably, a comparison of films on STO and LAO confirms that both the coercivity and the bias field of the former are significantly higher than those of the later. This strongly supports the theoretical analysis that a dominated rotation of *a*^−^ in tensile films contributes much to the exchange bias, whereas a mixture of tilting modes under compressive strain seriously causes an attenuation of exchange coupling, indicating a controllable exchange coupling by design of tilt pattern with epitaxial strain. Note that, the strain effect, which affects magnetocrystalline anisotropy, might contribute to the behavior of exchange bias, whereas it is negligible in our discussion for the small magnitude of anisotropy constant of both LSMO and SMO^29^,^30^.

For a further discussion, a prominent SMO thickness dependence of *H*_EB_ is demonstrated in Fig. 5c. As the bottom SMO layer becomes thicker, the strain in SMO at the interface is relaxed much more completely, resulting in a decline in the magnitude of the major tilting (Fig. 2d). Consequently, the interfacial effect, i.e. DM interaction, becomes dependent on the structure relaxation with the thickness. When the SMO thickness increases, *H*_EB_ of bilayers on STO is reduced significantly, taking the whole trend of the curve into account. However, the tendency for the LAO case is almost opposite. This difference could be understood in view of the different influence of strain relaxation on octahedral tilting and concomitant exchange coupling. For the scenario of tensile strain, the declining tilting angle of *a*^−^ naturally weakens the DM interaction and consequently the EB effect. Besides, the reducing difference in energy between *a*^−^ and *c*^−^ means a possible appearance of *c*^−^ with the relaxation, which would also attenuate the effective DM interaction and cause a decrease of *H*_EB_. The situation changes for SMO on LAO. According to Fig. 3a, b and Fig. 2d, relaxation of compressive strain triggers an increasing *a*^−^ tilting including both the ratio of *a*^−^/*c*^−^ and the tilting angle, responsible for the enhancing tendency of EB to SMO thickness. To be noted, an abnormal drop of *H*_EB_ happened when the SMO film on LAO became 40 u.c. thick. This might be attributed to the defects appearing at the interface with atomic scale roughness, when the thickness of SMO reaches 40 unit cell, which could be corroborated by the vanishment of RHEED oscillations^31^. The existence of defects would break the consistent contribution of *a*^−^ tilting and induce the decreasing trend of *H*_EB_. Despite a little imperfection, we see a quantitative way for tilt manipulation of exchange coupling via the thickness dependence of *H*_EB_.

### The quantitative effect of octahedral tilting on exchange bias

Finally, a simplified quantitative relationship between exchange bias and the magnitude of *a*^−^ tilting is given, providing a guidance for a better tilt engineering of exchange coupling. Calculated *H*_EB_ as a function of in-plane lattice constant is displayed in Fig. 5d, under a simplified assumption that *a*^−^ tilting is the only tilt pattern within the whole strain range. According to the sketch in Fig. 1e, interfacial Hamiltonian (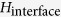) of *a*^−^ can be calculated with a simple sum:


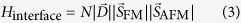


where *N* stands for the total number of interfacial Mn-O-Mn bonds. On the other hand, the relationship between *H*_interface_ and *H*_EB_ is given by


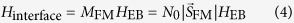


where *M*_FM_ parameters the saturation magnetization of the FM layer while *N*_0_ denotes the total number of FM atoms in the LSMO layer^32^. Thus, *H*_EB_ can be described by


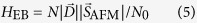



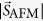
 is set to the calculated value of 2.52 μ_B_, while 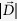 is calculated on the basis of Equation (2), with the displacement 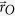 determined by lattice constant and the tilting angle in Fig. 2d. Here *γ* has a value of approximately 1 meV/Å for perovskites^18^. As can be seen from Fig. 5d, the maximum of *H*_EB_ is at the same level of magnitude with the experimental maximum value. Even they are quite close, ~1600 Oe and ~1000 Oe, respectively. The overestimation could be explained by the suppression of 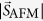 in SMO during the deposition compared to the theoretical value and the overestimated value of the effective tilting angle in the present case, where only uniaxial rotation is considered instead of the actual three-axis rotation in real system^33^. Note that the overestimation is enlarged for the compressive case. This behavior is ascribed to the failure of uniaxial tilting mode of *a*^−^ for the appearance of *c*^−^ and *c*^+^ under compressive strain. Besides, if the possible tilt pattern *a*^−^*a*^−^*a*^−^ of LSMO is taken into account^25^, the in-plane tilt pattern imprinted from LSMO to SMO in turn may cause an enhancement of *a*^−^ tilting and consequently the exchange bias within the whole strain range. Even so, a quantitative tilt engineering of exchange coupling is verified and becomes estimable according to the relationship between *H*_EB_ and tilting angle.

## Discussion

In conclusion, we reveal the indispensable role of *a*^−^ tilting in *G*-AFM-based exchange coupling system with DM interaction as a bridge. Based on the theoretical prediction, we switch the major tilt pattern in AFM layers between *a*^−^ and *c*^−^ by epitaxial strain and thus realize tilt-controllable exchange coupling experimentally in this *G*-AFM/FM system. Finally, a quantitative manipulation of exchange bias is demonstrated via controlling the magnitude of octahedral tilting at the interface, which would help not only the development of tilt engineering of magnetism, but also the application of tilt engineering in the field of multifunctional materials and AFM spintronics^34^,^35^.

## Method

### Sample preparation

All the samples were grown on the substrates with a (001)-surface and an edge orientation of [100], including SrTiO_3_ and LaAlO_3_. Pulsed laser deposition was used in our preparation to achieve atomic layer growth. The laser frequencies of 3 Hz and 7 Hz were used for the growth of SrMnO_3_ (SMO) and La_2/3_Sr_1/3_MnO_3_ (LSMO) respectively, providing an appropriate growth rate, about 1.2 nm/min. Substrate temperature was held at 790 °C for SMO and 680 °C for LSMO, while oxygen pressure was kept at the value of 35 mTorr and 50 mTorr for SMO and LSMO respectively, for an optimized layer-by-layer growth. Then samples cooled slowly down to room temperature under an oxygen pressure of 300 Torr for a further oxidation after deposition.

## Additional Information

**How to cite this article**: Li, F. *et al.* Tilt engineering of exchange coupling at *G*-type SrMnO_3_/(La,Sr)MnO_3_ interfaces. *Sci. Rep.* **5**, 16187; doi: 10.1038/srep16187 (2015).

## Supplementary Material

Supplementary Information
