# Supplementary material for: Tilt engineering of exchange coupling at G-type SrMnO3/(La,Sr)MnO3 interfaces
Source: Sci Rep. 2015 Nov 4;5:16187. doi: 10.1038/srep16187 (PMC4632028; doi:10.1038/srep16187)
Supplement: Supplementary Information [file srep16187-s1.doc]

**Supplemental Material**

**Tilt engineering of exchange coupling at *G*-type SrMnO3/(La,Sr)MnO3 interfaces**

F. Li, C. Song*, Y. Y. Wang, B. Cui, H. J. Mao, J. J. Peng, S. N. Li, G. Y. Wang, and F. Pan*

Key Laboratory of Advanced Materials (MOE), School of Materials Science and Engineering, Tsinghua University, Beijing 100084, China.

*E-mail: songcheng@mail.tsinghua.edu.cn; panf@mail.tsinghua.edu.cn.

**Details of first-principles calculations**

Projector augmented wave (PAW) implementation of the Vienna *ab initio* simulation package (VASP) was used in our work, within the generalized gradient approximation (GGA) method. Considering the doubling unit cell due to the spin configuration of SrMnO3, a 2 × 2 × 2 supercell was used, including 8 formula units with 40 atoms. We used an 8 × 8 × 8 Γ-centered k-point mesh and a cut-off energy of 550 eV for the plane-wave basis. Both the mesh and the energy have been increased until convergence.

**Calculations for energy of different spin configurations**

Considering the possible spin configuration of SrMnO3 in Fig. S1, i.e. FM (a), *A*-AFM (b), *C*-AFM (c) and *G*-AFM (d), we calculated the total free energy per formula unit with the in-plane lattice constant varying from 3.78 Å to 3.90 Å, corresponding to the lattice of LaAlO3 and SrTiO3 respectively, as demonstrated in Fig. S1(e). Note that *G*-AFM is energetically favorable within the whole strain range in our case, providing a basis for our discussion about exchange bias in *G*-AFM/FM heterostructures.


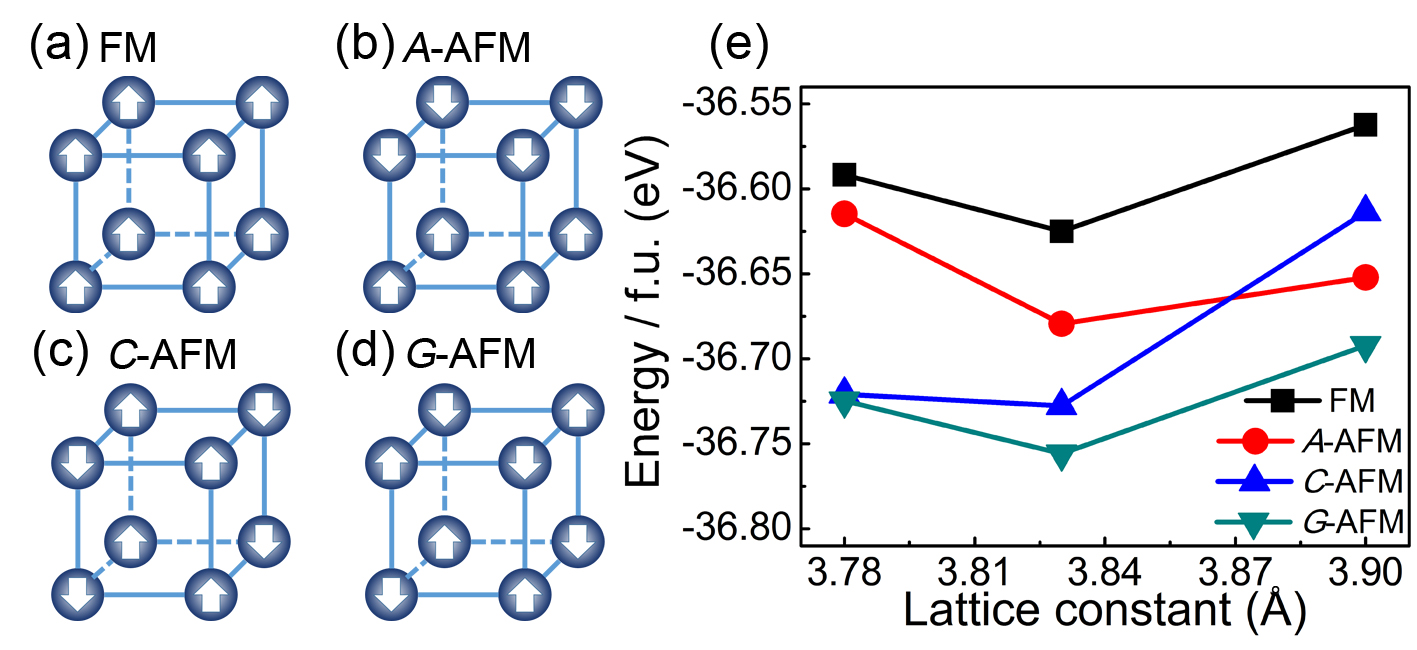


**Figure S1 | Strain dependent energy of different spin configurations for SMO.** Sketches of different spin configurations of SrMnO3: (a) FM, (b) *A*-AFM, (c) *C*-AFM and (d) *G*-AFM. (e) The total free energy per formula unit of SrMnO­3 as a function of the in-plane lattice constant for different spin configurations.

**RHEED during the deposition of SrMnO3/La2/3Sr1/3MnO3 bilayers**

Pulsed laser deposition was used in our preparation to achieve atomic layer growth. The process of growth was monitored by *in situ* RHEED (reflected high-energy electron diffraction). For example, for the preparation of SMO (20 u.c.)/LSMO (20 u.c.), the oscillating curve of RHEED is plotted in Fig. S2a, with the images of diffraction patterns at different time shown in Figs. S2b, S2c and S2d, corresponding to the bare substrate, the SMO single layer and the SMO/LSMO bilayer. The well-defined oscillation and clear diffraction spots and streaks imply a good layer-by-layer growth during the whole process and an atomic scale smoothness at the interface.


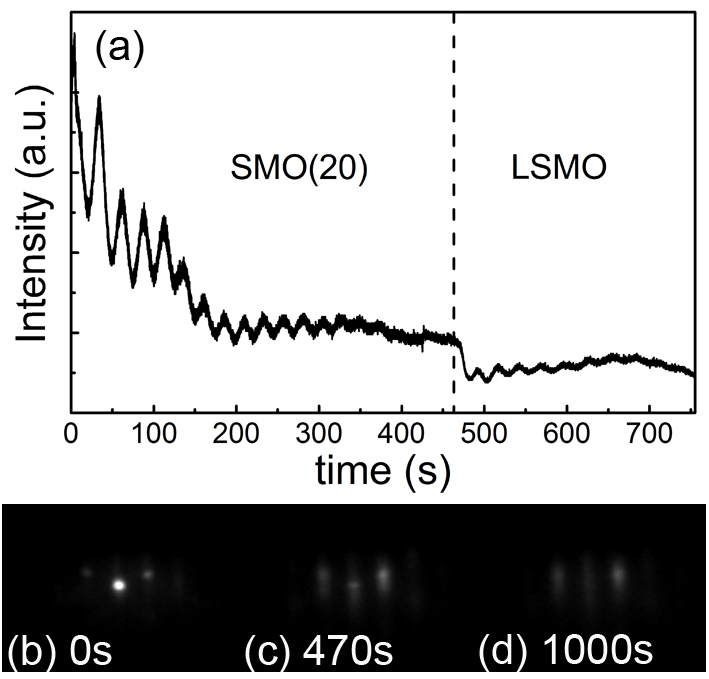


**Figure S2 | RHEED oscillating curve and diffraction patterns.** (a) The oscillating curve of RHEED during the preparation of the SrMnO3 (20 u.c.)/La2/3Sr1/3MnO3 (20 u.c.) bilayer. Images of diffraction patterns are captured at different growth time, corresponding to (b) the bare substrate, (c) the SMO single layer and (d) the SMO/LSMO bilayer.
